# Central mucoepidermoid carcinoma of the maxilla developing from a calcifying odontogenic cyst: A rare case report

**DOI:** 10.1002/ccr3.4928

**Published:** 2021-10-28

**Authors:** Megumi Isshiki‐Murakami, Hidetake Tachinami, Kei Tomihara, Akira Noguchi, Katsuhisa Sekido, Shuichi Imaue, Kumiko Fujiwara, Johji Imura, Makoto Noguchi

**Affiliations:** ^1^ Faculty of Medicine Department of Oral and Maxillofacial Surgery Academic Assembly University of Toyama Toyama Japan; ^2^ Division of Oral and Maxillofacial Surgery Niigata University Graduate of School of Medical and Dental Sciences Niigata Japan; ^3^ Faculty of Medicine Department of Diagnostic Pathology Academic Assembly University of Toyama Toyama Japan; ^4^ Department of Oral and Maxillofacial Surgery Toyama Red Cross Hospital Toyama Japan

**Keywords:** calcifying odontogenic cyst, intraosseous, maxilla, mucoepidermoid carcinoma, salivary gland tumor, young adult

## Abstract

Intraosseous mucoepidermoid carcinoma of the jaw is a rare lesion that has been suggested to originate from the odontogenic epithelium. We report an unusual case of central mucoepidermoid carcinoma in an 18‐year‐old Japanese man with an odontogenic cyst.

## Graphical abstract

This report describes the clinical and histologic features of this extremely rare case of central mucoepidermoid carcinoma admixed with a calcifying odontogenic cyst.

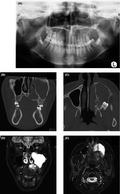

## 1 INTRODUCTION

Mucoepidermoid carcinoma (MEC) is the most common malignant salivary gland tumor, accounting for 20% of all malignant salivary gland tumors.^1^

The sites most often affected by MEC are the parotid gland or the minor salivary glands of the palate, retromolar area, and lip.^1^ Intraosseous involvement of the jaws (central) has been reported as a rare type of this lesion, comprising 2%–4.3% of all MECs reported, and is more prevalent in the mandible than in the maxilla.^2^ Central MEC (CMEC) among young patients (aged under 20 years) is extremely rare, and only 13 cases of CMEC of the maxilla have been reported in the English language literature (Table 1).^3^, ^4^, ^5^, ^6^, ^7^, ^8^, ^9^, ^10^, ^11^, ^12^, ^13^, ^14^, ^15^

**TABLE 1:** Central mucoepidermoid carcinoma of the maxilla in patients younger than 20 years of age

Case	Author, year	Age, gender	Symptoms	Treatment	Grade	Prognosis
1	Hertz, 1952^3^	15/F	NA	Curettage	NA	Dead at age 16
2	Chaundry et al., 1961^4^	16/F	NA	Maxillectomy	NA	Healthy after 3 yr
3	Bronwand et al., 1975^5^	15/M	NA	Maxillectomy	Low	Healthy after 12 yr
4	Namin et al., 2005^6^	11/F	Swelling	Maxillectomy	High	NA
5	Moraes P, et al 2007^7^	14/F	Swelling	Maxillectomy	Low	NED at 4 yr
6	He Y, Wang J, et al 2012^8^	19/F	Numbness, odontoseisis	Maxillectomy	Intermediate	NED at 28 mo
7	Takano et al 2012^9^	18/M	Swelling	Cystectomy followed by partial maxillectomy		NED at 2 yr
8	Zhou et al, 2012^10^	15/F	Swelling	Curettage	Low	Recurrence at 60 mo, NED at 91 mo
9	Rathore AS, et al 2014^11^	18/M	Swelling	Maxillectomy with neck dissection	Low	NED at 1 yr
10	Kim SM, et al 2015^12^	11/M	Swelling	Hemimaxillectomy	Low	NED at 3 yr
11	Del Corso G, et al 2016^13^	16/F	Swelling	Maxillectomy	Internmediate	NED at 6 yr
12	Bell D, et al 2016^14^	17/F	NA	Surgery	Internmediate	NED at 44 mo
13	Bell D, et al 2016^14^	8/F	NA	Surgery with PORT	High	NED at 8 mo
14	Present case	18/M	Swelling	Cystectomy followed by partial maxillectomy	Low	NED at 2 yr

Abbreviations: F, female; M, male; mo, months; NA, not available; NED, no evidence of disease; PORT, post‐operative radiotherapy; yr, years.

We report an additional case of CMEC of the maxilla in an 18‐year‐old Japanese man. Pathological examination revealed that the lesion was admixed with a calcifying odontogenic cyst. The odontogenic epithelium of the calcifying odontogenic cyst was highly likely to be the origin of this case. To the best of our knowledge, this is the first report of CMEC of the maxilla associated with a calcifying odontogenic cyst. We report the case of MEC arising from a rare location, with distinct radiographic findings, and discuss the etiology of the lesion with reference to the relevant literature.

## 2 CASE REPORT

**FIGURE 1:**
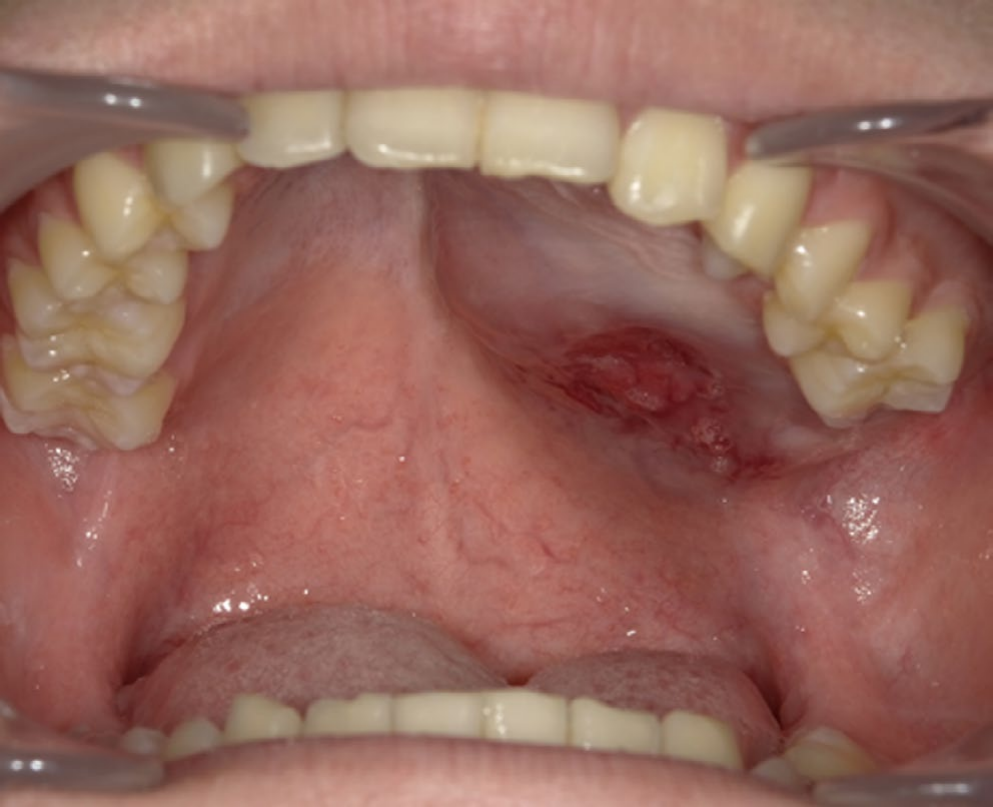
An intraoral photograph showing a swelling of the left side of the hard palate with a partial ulceration

**FIGURE 2:**
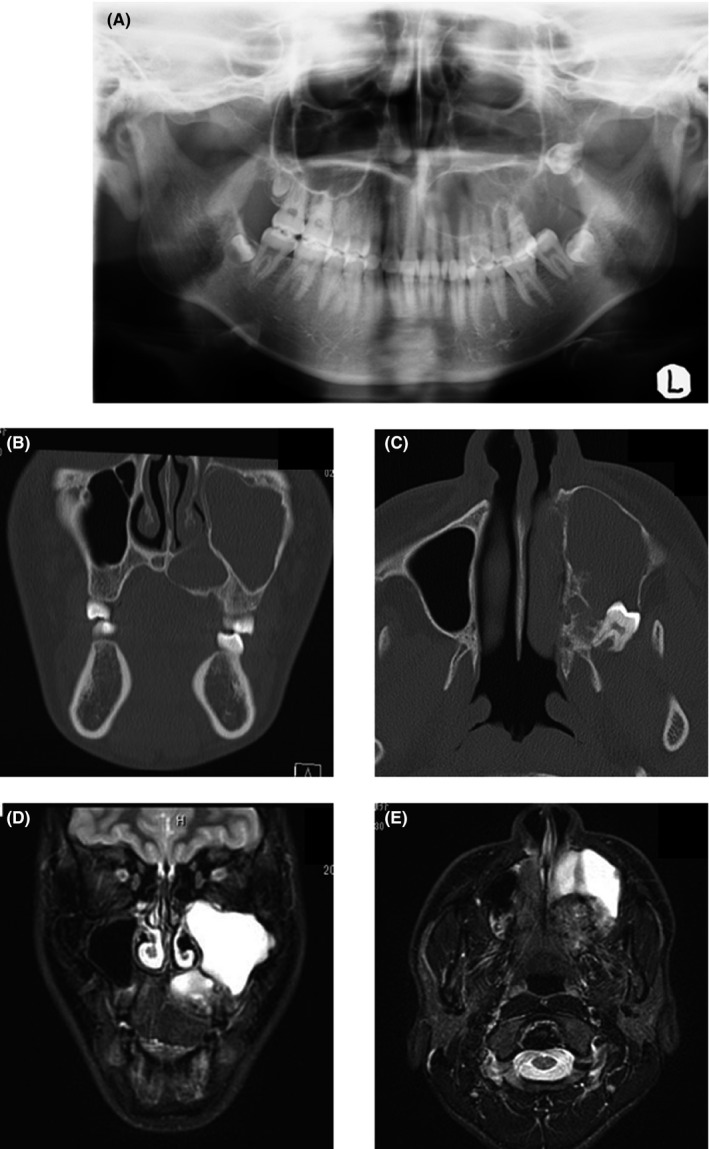
(A) Panoramic radiograph showing radiopacity of the left maxillary sinus with an impacted tooth and descent of the maxillary sinus floor. (B) Coronal view of computed tomography (CT) scan showing multilocular radiolucent expansile area of the left maxillary sinus. (C) Axial view of CT scan showing expanding lesion without bony perforations of the outer cortex of the left maxillary sinus accompanied by an impacted tooth in the posterior wall. (D) Coronal view of a magnetic resonance imaging (MRI) showing a nonhomogeneous mild‐enhancing area in the left hard palate and alveolar process and the highly enhancing homogeneous area in the left maxillary sinus. (E) Axial view of an MRI

An 18‐year‐old man was referred to our hospital in 2019 with a complaint of a painless mass in the left palate. The patient was aware of the swelling in his right palate for approximately 2 years. However, he noticed a recent and rapid increase in the swelling. Hence, the patient initially consulted a general dental practitioner, where a diagnosis of a tumor of the palate was established. The patient was subsequently referred to our department. His medical history revealed febrile seizures at the age of 1 year, and his family history was unremarkable. On initial assessment, no systemic symptoms were observed. An extra‐oral examination revealed facial symmetry. No swollen cervical lymph nodes were palpable bilaterally. Intraoral examination revealed a dome‐shaped mass measuring 4.0 × 3.0 cm on the left side of the hard palate, extending anteriorly to the distal first premolar area and posteriorly to the soft palate. The mucosal surface of the mass was smooth, but partially ulcerated (Figure 1). On palpation, the mass was elastic hard in consistency. Panoramic radiography revealed opacity of the left maxillary sinus with an impacted tooth and the level of the left maxillary sinus floor was lower than that on the right side (Figure 2A). Computed tomography (CT) revealed a well‐demarcated multilocular‐appearing expansile radiolucent lesion in the left maxillary sinus with an impacted tooth in the posterior wall (Figure 2B,C). Magnetic resonance imaging revealed a nonhomogeneous, mildly enhancing lesion in the left hard palate and alveolar process, along with a highly enhancing homogeneous lesion in the left maxillary sinus (Figure 2D,E). CT of the neck revealed no enlarged bilateral lymph nodes, and CT of the abdominal and thoracic regions revealed no other specific findings.

**FIGURE 3:**
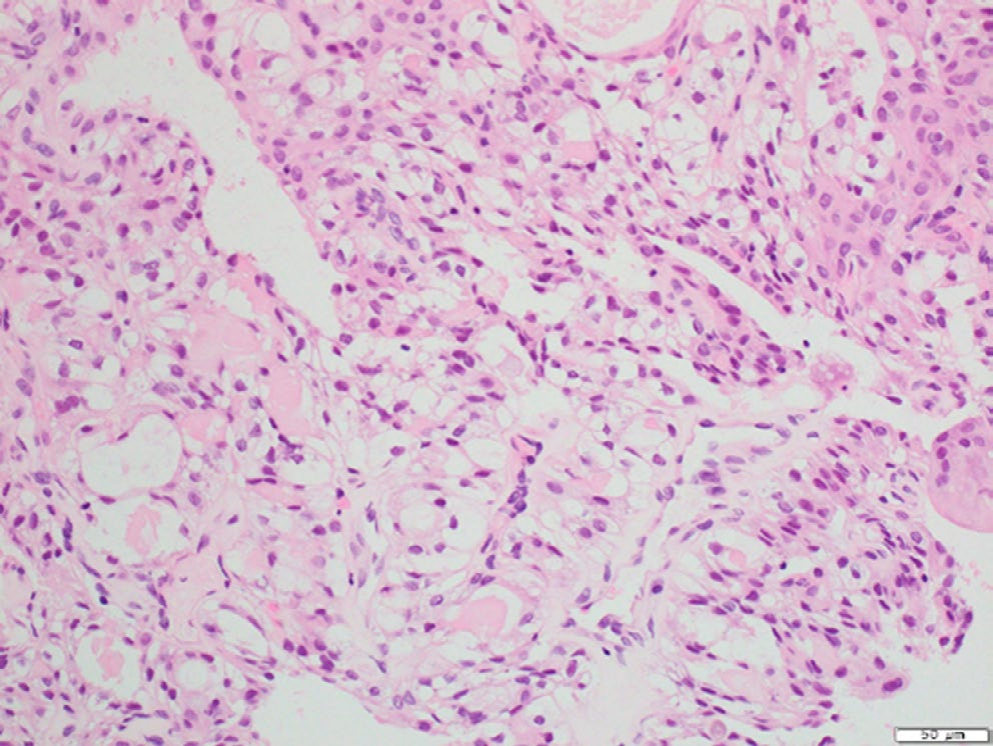
Histological appearance of the biopsy specimen (hematoxylin–eosin stain). Photomicrograph of the histological specimen showing the epithelium composed of primarily squamous epithelial cells and occasionally clear cells and ghost cells

**FIGURE 4:**
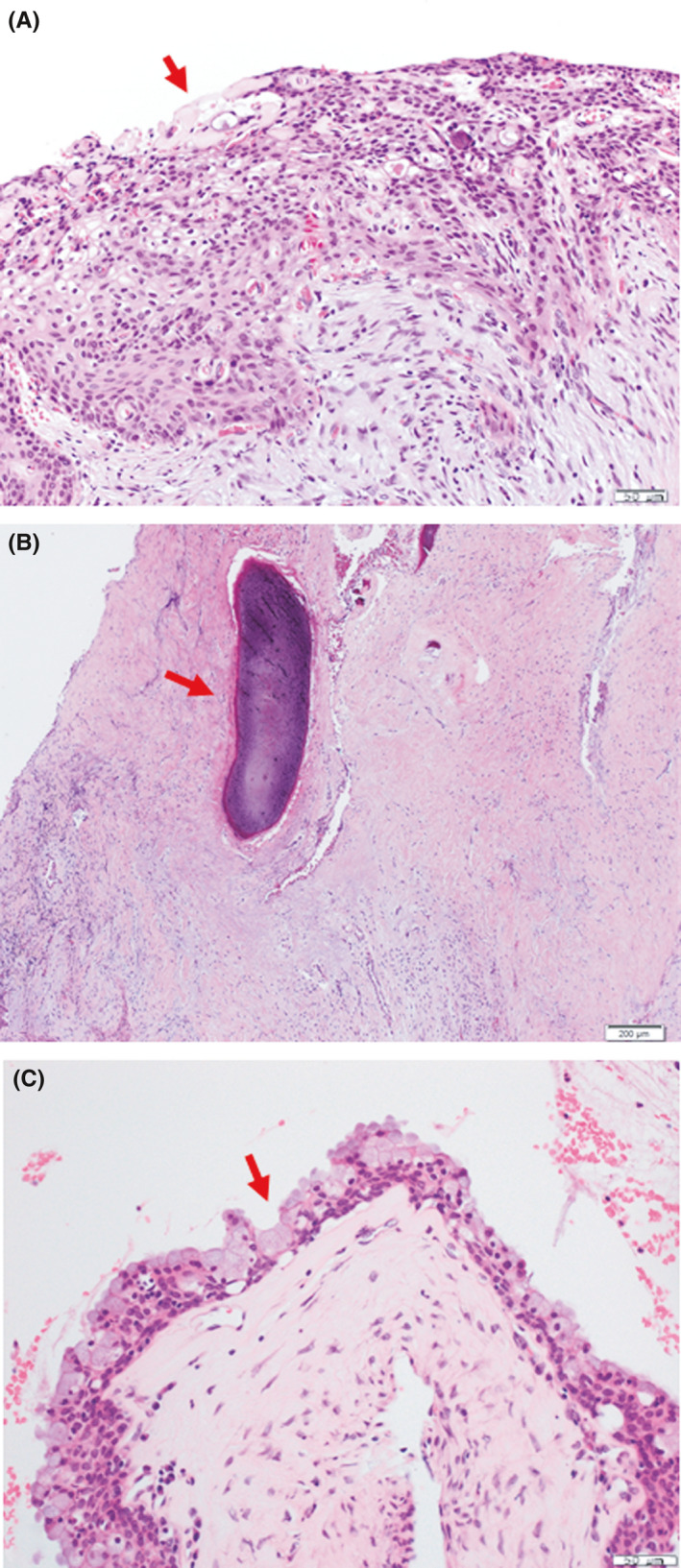
Histological findings of the cystic lesion occupying the left maxillary sinus (hematoxylin–eosin stain). (A) The lesion showing the odontogenic epithelium which is composed of primarily stratified squamous epithelial cells with the focal presence of calcification (arrows). (B) Dentinoid (arrows). (C) Mucin‐producing cells (arrows) in the epithelium

**FIGURE 5:**
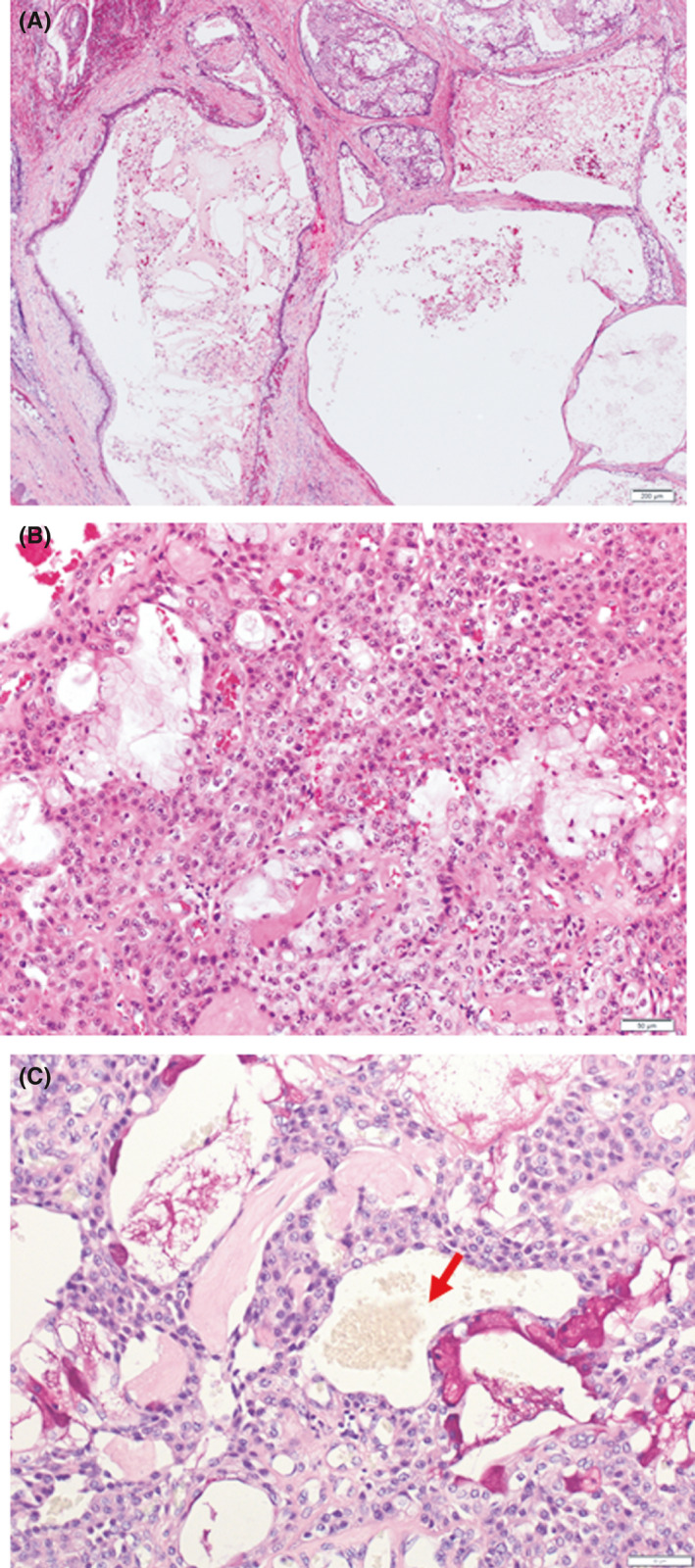
Histological appearance of the tumor (hematoxylin–eosin stain). (A) A low‐power view of the tumor showing multiple cystic structures admixed with carcinomatous components in the cystic space. (B) A high‐power view of the carcinomatous component showing abundant intermediate cells and mucin‐producing cells. (C) Mucin production (arrow)

Incisional biopsy of the palate revealed the epithelium comprising primarily squamous epithelial cells and the focal presence of clear cells and ghost cells along with the presence of calcifications, which suggested the possibility of benign odontogenic tumors or odontogenic cysts (Figure 3). Based on these pathological findings and the presence of an impacted tooth, calcifying epithelial odontogenic tumor or calcifying odontogenic cyst was considered as a differential diagnosis. Surgical resection of the cystic lesion occupying the maxillary sinus and removal of the impacted tooth were performed under general anesthesia followed by open packing of the residual osseous defect, and the excised specimen was subjected to further examination for pathological diagnosis. Microscopically, multiple cystic lumina lined by odontogenic epithelium were observed. The epithelium primarily comprised stratified squamous epithelial cells with the focal presence of clear cells, ghost cells, dentinoid, and calcification, suggesting a diagnosis of calcifying odontogenic cyst (Figure 4). However, because heterotopic mucin‐producing cells were observed in the odontogenic epithelium, the possibility of accompanying lesions, such as MEC, was considered. Therefore, en bloc resection by partial maxillectomy from the first premolar to the second molar, palatal bone, and alveolar process was performed. Macroscopically, the surgical specimen consisted of white‐gray solid areas within the alveolar process and hard palate of the cut surface. Microscopically, the tumor was poorly circumscribed and not completely encapsulated. The tumor comprised multiple cystic lumina of various sizes lined by odontogenic epithelium, and these cystic spaces were filled with carcinomatous components, which primarily comprised abundant intermediate cells and mucin‐producing cells (Figure 5A,B). Mucicarmine stain demonstrated mucin production (Figure 5C). Nuclear atypia was slight and mitotic figures were rare in carcinomatous components. Moreover, the palatal salivary glands were intact. These histopathological features led to the diagnosis of intraosseous low‐grade MEC of the maxilla arising from a calcifying odontogenic cyst. The surgical defect following partial maxillectomy was rehabilitated with an obturator, and no evidence of local recurrence or distant metastasis was found at the 19‐month follow‐up after surgery.

## 3 DISCUSSION

To the best of our knowledge, this is the first report of a central mucoepidermoid carcinoma (CMEC) of the maxilla arising from a calcifying odontogenic cyst.

Mucoepidermoid carcinoma is the most common malignant salivary gland tumor, accounting for 20% of all malignant salivary gland tumors mainly observed in the parotid gland of the major salivary gland and around 30% observed in minor salivary glands, including the palate, lip, and retromolar area.^1^ MEC rarely occurs in the jaw bones (central) and is considered a rare variant of MEC.^2^ However, because of its rarity and inconsistency through individual case reports, only limited information regarding its clinical and biological behaviors, pathogenesis, treatment, and prognosis is available.^15^, ^16^

In 2012, as a first large‐scale study, Zhou et al. reported that in 39 Chinese patients diagnosed with CMEC from 1985 to 2010, the lesion was more prevalent in women than in men, highly prevalent in the fourth to fifth decades of life, and more prevalent in the mandible than in the maxilla. Moreover, according to this study, the most common symptoms were asymptomatic swelling accompanied by the most common radiographic unilocular or multilocular and osteolytic radiolucent lesions. Furthermore, the most frequent histologic type was low‐grade CMEC with favorable prognosis.^10^ More recently, de Souza et al. reviewed 36 publications with 147 cases of CMEC and performed a comprehensive analysis of CMEC's clinical and biological characteristics. According to this literature review, the lesion was relatively more prevalent in women (51.7%) than in men (48.3%) and more prevalent in the mandible (93 cases, 63.3%) than in the maxilla (54 cases, 36.7%). Although the mean age of patients with CMEC was 46.51 (range, 11–79) years and CMEC was highly prevalent in the fifth to seventh decades of life, 51 patients (34.7%) were younger than 40 years and 14 patients (10%) were younger than 20 years.^17^ Most of the CMEC cases were histologically classified as low‐grade (54.4%) and usually had a favorable prognosis, whereas local recurrence and distant metastasis were observed in 16 patients (10.88%) and 3 patients (2.0%), respectively, and 11 of these 16 cases of local recurrence and 2 of these 3 cases of distant metastasis were of low‐grade.^17^

Although the origin of CMEC of the maxilla remains controversial, various theories have been considered.^18^ Neoplastic transformation of heterotopic salivary gland tissue in the jaw bones, the epithelial lining of the maxillary sinus, and odontogenic cysts are considered to be the likely risk of the CMEC, although direct evidence for such pathogeneses has not been well documented in previous reports so far. In our case report, heterotopic mucin‐producing cells were observed in the odontogenic epithelial lining of the cyst wall that may very likely explain the pathogenesis of CMEC.

Because the clinical finding of CMEC of the maxilla may resemble benign lesions such as odontogenic cysts, tumors, or odontogenic infectious diseases, it may be difficult to establish a correct diagnosis initially. Our case report also showed a radiolucent cystic lesion with an impacted tooth, which is a typical radiographic finding of a benign odontogenic cyst or tumor. Therefore, initially, we did not consider the possibility of MEC. However, in our case report, the maxillary radiolucent lesion was accompanied by proliferative and destructive bone lesion in the hard palate and alveolar process. Considering these findings, CMEC or other central malignant tumors should have been considered as a differential diagnosis even if clinical and radiographic findings lacked the features of malignancy.^19^

In conclusion, we report an extremely rare case of CMEC of the maxilla arising from a calcifying odontogenic cyst in a young patient. Because information regarding the clinical characteristics of CMEC in young adults is limited, long‐term follow‐up would be desirable considering the possibility of late local recurrence and metastasis. Clinical findings of CMEC in the maxilla may cause confusion during the initial diagnosis considering that CMEC is similar to benign lesion, as in our case report. Therefore, caution should be exercised regarding CMEC in the maxilla regardless of patient's age to establish an appropriate early diagnosis and treatment even if the clinical findings are not suggestive of malignancy.

## CONFLICT OF INTEREST

The authors made no disclosures.

## AUTHOR CONTRIBUTIONS

KT, HT, and MN contributed to the manuscript preparation. MIM, HT, KS, SI, and KF contributed to the patient management. AN and JI are pathologists who contributed to the pathological diagnosis. All authors read and approved the final manuscript.

## ETHICAL APPROVAL

Because this report involves no experiment, ethics approval is waived.

## CONSENT

A written informed consent was obtained from the patient for publication of this case report and any accompanying images. A copy of the written consent is available for review by the Editor of this journal.

## Data Availability

Data sharing is not applicable to this article as no new data were created or analyzed in this study.
